# Impact of Antibiotic Stewardship on Treatment of Hospitalized Children with Skin and Soft-Tissue Infections

**DOI:** 10.3390/children11111325

**Published:** 2024-10-30

**Authors:** Giulia Brigadoi, Sara Rossin, Lorenzo Chiusaroli, Giulia Camilla Demarin, Linda Maestri, Francesca Tesser, Martina Matarazzo, Cecilia Liberati, Elisa Barbieri, Carlo Giaquinto, Liviana Da Dalt, Silvia Bressan, Daniele Donà

**Affiliations:** 1 Division of Pediatric Infectious Diseases, Department of Women’s and Children’s Health, Padua University Hospital, 35128 Padua, Italy; giulia.brigadoi@phd.unipd.it (G.B.);; 2 Pediatric Emergency Department, Department of Women’s and Children’s Health, Padua University Hospital, 35128 Padua, Italy

**Keywords:** antibiotic stewardship programs, ASPs, children, skin and soft-tissue infections, SSTIs, antibiotics

## Abstract

Background: Skin and soft-tissue infections (SSTIs) are common infectious syndromes in children. Overusing broad-spectrum antibiotics has contributed to rising antibiotic resistance, complicating treatment outcomes. To address this issue, antimicrobial stewardship programs (ASPs) have been implemented to optimize antibiotic use. This study evaluated the impact of a multifaceted ASP on antibiotic prescribing practices for SSTIs in a pediatric acute care setting over eight years. Methods: We conducted a quasi-experimental study at the Pediatric Acute Care Unit of the Padua University Hospital, including children admitted with SSTIs from October 2014 to September 2022, to evaluate the impact of a multifaceted ASP implemented in October 2015. The study was divided into three periods: pre-implementation (October 2014–September 2015), post-implementation (October 2015–March 2020), and COVID-19 (April 2020–August 2022). Data on antibiotic prescriptions and microbiological results were collected and analyzed. Results: The implementation of the ASP led to a significant reduction in the use of broad-spectrum antibiotics, particularly third-generation cephalosporins (from 40.4% to 9.8%) and glycopeptides (from 21.1% to 1.6%). There was a notable increase in the prescription of Access antibiotics, from 30% in the pre-implementation to over 60% in the post-implementation and 80% during COVID-19. No increase in the hospital length of stay was observed. Microbiological results showed no significant changes in bacterial profiles over time. Conclusions: The use of the ASP effectively improved antibiotic prescribing practices, reducing reliance on broad-spectrum antibiotics even during the COVID-19 pandemic. These findings highlight the value of ongoing stewardship efforts and suggest the need for similar programs in ambulatory settings to further address antibiotic resistance.

## 1. Introduction

Skin and soft-tissue infections (SSTIs) are among the most common infectious conditions in children, with a wide range of presentations, from mild to severe [1]. These infections affect the skin and surrounding tissues and may sometimes extend to the subcutaneous layers. The most common pathogens responsible for skin infections in children are bacteria, particularly Gram-positive bacteria like *Staphylococcus aureus* and *Streptococcus pyogenes*. However, Gram-negative bacteria can also cause SSTIs [2].

While most SSTIs can be managed on an outpatient basis with topical or oral antibiotic treatments, parenteral antibiotics may be required in some cases [3]. The choice of antibiotics for SSTI treatment should be guided by local epidemiology. Broad-spectrum antibiotics are recommended for methicillin-resistant *S. aureus* (MRSA) infections in areas with high resistance prevalence or in patients at greater risk, such as those with a history of hospital admission or recurrent, hard-to-treat infections [4,5].

However, the overuse of broad-spectrum antibiotics, especially when not necessary, poses a serious challenge [6]. Often, this choice is driven by concerns over potential complications from using narrow-spectrum therapies, as well as by insufficient knowledge of the local epidemiology and resistance mechanisms [7]. The excessive and inappropriate use of antibiotics has contributed significantly to the rise of antimicrobial resistance (AMR). While the development of resistance can be an intrinsic bacterial mechanism and occur naturally, the misuse of antibiotics accelerates the selection of resistant organisms, increasing selective pressure on the global microbial population and fostering the emergence of resistant strains [8].

In response to the growing threat of antimicrobial resistance, the Infectious Diseases Society of America (IDSA) introduced the concept of Antimicrobial Stewardship Programs (ASPs) in 2007 [9]. ASPs constitute a set of interventions or strategies designed to optimize antibiotic use, guiding the selection of appropriate antibiotics, dosages, and durations in various situations. These programs aim to limit selective pressure on bacterial strains and reduce the potential adverse effects of antibiotic therapy without compromising patient outcomes. ASPs have proven effective in reducing antibiotic prescriptions in both hospital and community settings across the United States and Europe [10].

Moreover, in order to identify antibiotics that are priorities for monitoring and surveillance, and those with the potential to induce and propagate resistance, the WHO Expert Committee established the Access, Watch, and Reserve (AWaRe) classification in 2017 [11]. This classification has been revised over the years, with new antibiotics added, and the most recent version was published in 2023. Antibiotics are categorized into three groups based on their spectra of activity, risks of side effects, potential for resistance development, and costs to emphasize the importance of their appropriate use. The Access category includes 87 antibiotics such as amoxicillin, amoxicillin-clavulanic acid, and clindamycin. The Watch category includes 141 antibiotics such as ceftriaxone, vancomycin, teicoplanin, and meropenem. The Reserve category contains 29 antibiotics including linezolid and daptomycin. Antibiotics in the Access category should be prioritized as first-line treatments whenever possible, and global supply should be sufficient to meet demand. The WHO AWaRe classification recommends the use of amoxicillin-clavulanate, cephalexin, or cloxacillin, all of which belong to the Access category, for the treatment of SSTIs in children. Our study sought to evaluate the impact of a multifaceted ASP on antibiotic selection in children admitted to a pediatric acute care ward for SSTIs over an eight-year period.

## 2. Materials and Methods

### 2.1. Setting and Study Design

This quasi-experimental study with retrospective data collection was conducted at the Pediatric Acute Care Unit (PACU) within the Department of Women’s and Children’s Health at Padua University Hospital in Italy. Our Children’s Hospital offers primary and secondary care to a metropolitan population of 350,000 people, including 45,000 children under the age of 15. It also provides tertiary care to both regional and extra-regional populations, with an annual volume of 25,000 to 27,000 visits to the Pediatric Emergency Department.

### 2.2. Population and Study Period

The study included all the children admitted to the PACU from the Pediatric Emergency Department between 1 October 2014 and 30 September 2022 with a clinical diagnosis of SSTIs or burns who received at least one systemic antibiotic prescription during their admission. Children receiving only topical antibiotic treatment for SSTI and those transferred from other hospitals or wards were excluded from the study.

### 2.3. Intervention

Since October 2015, a multifaceted and multistep ASP has been implemented in both the emergency department and the pediatric acute care unit. This program introduced several interventions for managing infectious diseases. The first intervention involved implementing clinical pathways for managing the most common infectious syndromes to guide clinicians in deciding both whether to prescribe antibiotics as well as which antibiotics to use, at what dosage, and for how many days. Additionally, all internal guidelines were updated based on the latest evidence, offering physicians evidence-based recommendations. These interventions were complemented by educational sessions led by pediatric infectious disease specialists, aiming to provide knowledge on epidemiology, etiology, resistance mechanisms, and antibiotic selection for common pediatric infections. These lessons had been designed for both residents and senior doctors and were repeated every two years due to the high turnover of residents in the hospital. Lastly, a pediatric infectious disease physician began participating in ward rounds twice weekly, or more frequently in complex cases, to discuss antibiotic treatment for patients admitted to PACU with the team.

For SSTI management, an updated protocol and specific training sessions were introduced between 2015 and 2016, offering new recommendations for both outpatient and inpatient care. All the antibiotics recommended as first-line therapy belong to the Access category. For inpatient treatment, amoxicillin-clavulanate, first-generation cephalosporins, and oxacillin were recommended as first-line treatments while clindamycin was advised for suspected MRSA infections. The use of third-generation cephalosporins (e.g., ceftriaxone) in combination with glycopeptides (teicoplanin or vancomycin) was reserved for severe infections, immunocompromised patients, or suspected bacteremia or for children with treatment failure with first-line treatment.

To evaluate the impact of the ASP and to assess whether the COVID-19 pandemic—which temporarily interrupted all educational sessions and multidisciplinary meetings, followed by a shift to virtual lessons—affected antibiotic prescribing practices in the PACU, the study period was divided into three periods:

- Pre-implementation period: from October 2014 to September 2015, before the ASP was introduced.
- Post-implementation period: from October 2015 to March 2020, during the ASP implementation.
- COVID-19 period: from April 2020 to August 2022.

### 2.4. Data Source and Collection

Clinical, demographic, diagnostic, and daily prescription data were manually extracted by medical doctors from electronic medical records using a password-protected REDCap data collection form. To guarantee anonymity and privacy, a unique study-specific number was assigned to each patient. For each patient, prescription data included the type of antibiotic, route of administration, dosage, therapy start and end dates, and the reasons for any interruption or modification of therapy. Additionally, microbiological data from blood cultures, skin swab cultures, and purulent material cultures were collected when available. Pus and exudate samples were collected into dry sterile bottles when possible, or, if not, a swab was taken, ensuring the deepest part of the wound was sampled rather than the superficial flora. Bacterial swabs with transport medium for routine culture were used and all specimens were analyzed using standard culture methods. All tests were conducted as part of routine clinical practice, with no additional tests performed for research purposes. Data for patients transferred to another ward or hospital were limited to their admission to the Pediatric Acute Care Unit (PACU).

### 2.5. Outcomes

The primary outcome of the study was to assess the change in antibiotic prescription rates following the introduction of the ASP, classifying antibiotics prescribed based on the AWaRe classification. Moreover, we assessed the changes in antibiotic prescriptions by analyzing the specific types of antibiotics prescribed, stratified by class and, where applicable, by generation. This included penicillins (e.g., oxacillin), aminopenicillins (e.g., amoxicillin-clavulanic acid and ampicillin-sulbactam), lincosamides (e.g., clindamycin), first-generation cephalosporins (e.g., cephalexin and cefazolin), third-generation cephalosporins (e.g., ceftriaxone, cefotaxime, and ceftazidime), glycopeptides (e.g., vancomycin and teicoplanin), and other antibiotics (e.g., trimethoprim-sulfamethoxazole, levofloxacin, and other less frequently used antibiotics not included in the previously mentioned classes).

The analyses were conducted considering the total hospital antibiotic prescription (parenteral and oral) and the oral antibiotic prescriptions after parenteral therapy (both if started in the hospital and completed at home or started at the time of discharge). For the primary outcome, the analysis was conducted on the total population included in the study. Secondary outcomes included evaluating the changes in the days of therapy (DOT), length of therapy (LOT), and the ratio DOT/LOT considering the antibiotic therapy prescribed in hospital and the complete antibiotic course (hospital plus antibiotic therapy at home). For this analysis, children transferred to other wards or hospitals before the end of the treatment were excluded. Other secondary outcomes considered were the change in the length of hospital stay, the rate of side effects reported related to antibiotics, and the epidemiology and correlation between broad-spectrum antibiotic use and microbiological evidence of multidrug-resistant pathogens.

### 2.6. Statistical Analysis

The analyses were descriptive in nature. Results from the different study periods were summarized as numbers and percentages for categorical variables and as medians with 25th and 75th interquartile ranges for continuous variables. Categorical variables were compared with χ2 or Fisher’s 2-tailed exact test in a contingency table and continuous variables with the non-parametric Kruskal–Wallis rank-sum test due to the non-normal distribution of data. A two-sided *p* < 0.05 was considered statistically significant. Antibiotic prescription data were analyzed by stratifying based on the three defined periods and further subdivided into 16 six-month intervals. Microbiological results were also analyzed by stratification across the same three periods. All analyses were conducted using R statistical software, version 2023.12.1 (R Foundation for Statistical Computing, Vienna, Austria), and Microsoft Excel for Mac, version 16.68.

## 3. Results

### 3.1. Demographic Characteristics and Diagnosis of Skin and Soft-Tissue Infections

During the study period, 6521 children were admitted from the pediatric emergency department to the PACU, of which 3023 (3023/6521, 46.4%) received at least one antibiotic prescription. Of these, 184 (184/3023, 6.1%) children were admitted with a diagnosis of SSTI: 27 during the pre-implementation period, 117 during the post-implementation period, and 40 during the COVID-19 period. The median age was consistent across the three groups (median age 54.1 months, IQR 18.6–109.4), with a higher prevalence of males (110/184, 59.8%). The population characteristics are summarized in Table 1.

Cellulitis and impetigo were the most common reasons for admission, accounting for 64.1% of cases (119/184), while skin abscesses and complicated SSTIs, such as pyomyositis and fasciitis, were less common. The remaining types of infections represented a small percentage of the total cases (see Table 2).

### 3.2. Antibiotic Prescription in Hospital

**Figure 1:**
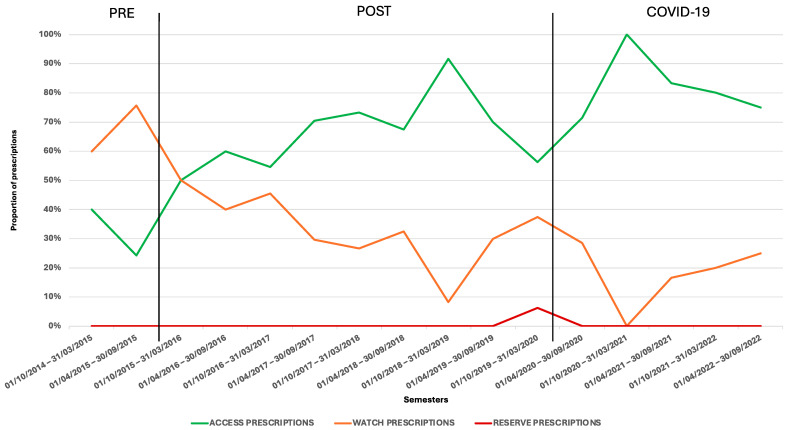
Antibiotics prescribed for SSTI management in the different semesters during hospital admission based on the AWaRe classification.

A total of 317 antibiotic prescriptions in the hospital, both oral and parenteral, were analyzed over the eight-year study period. The use of Watch antibiotics decreased significantly over time, from nearly 80% in the second semester of the pre-implementation period to less than 40% during the post-implementation period, with this reduction sustained throughout the COVID-19 period (Figure 1, Table S1) (*p* < 0.00001). Conversely, the prescription of Access antibiotics, which was relatively low during the pre-implementation period (less than 40% of total prescriptions), gradually increased following the ASP implementation, reaching more than 60% of total prescriptions in nearly all semesters of the post-implementation period and further rising to 80.0% during the COVID-19 period (*p* < 0.00001). Reserve antibiotics were prescribed only once during the post-implementation period.

**Figure 2:**
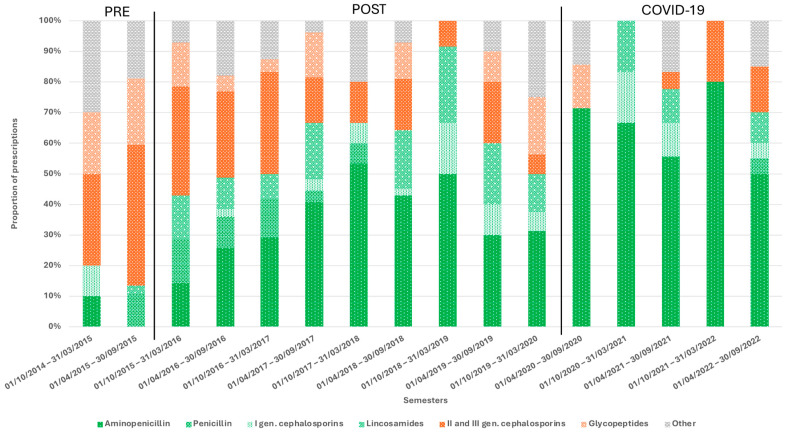
Types of antibiotics prescribed for SSTI management during hospital admission in the different semesters.

In the pre-implementation period, third-generation cephalosporins combined with glycopeptides were the most commonly prescribed antibiotics, accounting for 61.4% of all prescriptions (35/57). In contrast, aminopenicillins were rarely used. Following the implementation of the ASP, there was a progressive statistically significant increase in the use of aminopenicillins (especially amoxicillin-clavulanate) and lincosamides (clindamycin) (*p* < 0.00001 and *p* = 0.023, respectively), particularly in patients at higher risk for MRSA infections, with these antibiotics comprising 68.9% of total prescriptions during the COVID-19 period (42/61). Concurrently, there was a notable reduction in the use of third-generation cephalosporins (e.g., ceftriaxone) and glycopeptides (e.g., teicoplanin and vancomycin) (*p* < 0.001 and *p* = 0.002, respectively), with glycopeptide prescriptions nearing zero during the COVID-19 period (Figure 2, Table S2).

### 3.3. Switch from Parenteral to Oral Therapy

**Figure 3:**
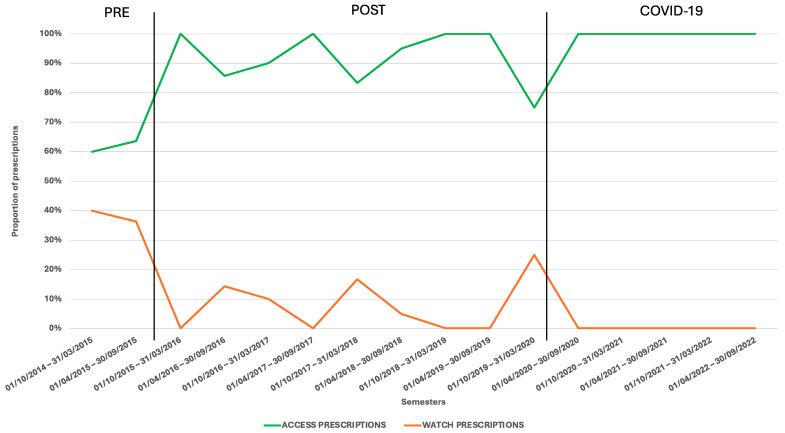
Oral antibiotics prescribed for SSTI management in the different semesters after parenteral therapy based on the AWaRe classification.

The numbers of patients receiving an oral antibiotic prescription after parenteral therapy were similar in the three periods. Considering antibiotics based on the AWaRe classifications, in the pre-implementation period, 37.5% (6/16) of prescriptions belonged to the Watch category. In the semesters post the implementation of ASP, an increase in the use of Access antibiotics and a reduction in the use of Watch antibiotics were observed (*p* = 0.0004). In the COVID-19 period, all the children with oral antibiotic prescriptions were treated with Access antibiotics (Figure 3, Table S3).

**Figure 4:**
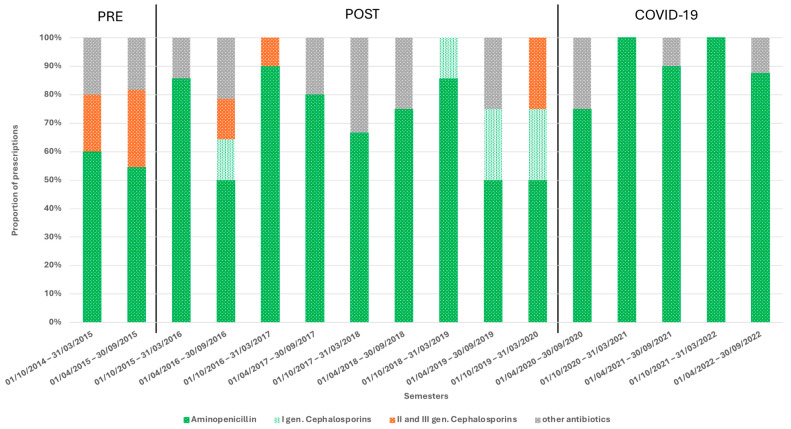
Types of antibiotics prescribed for SSTI management after parenteral therapy in the different semesters.

Aminopenicillin, especially amoxicillin-clavulanic acid, emerged as the most commonly prescribed oral antibiotic. In the initial semesters, second- and third-generation cephalosporins (e.g., cefuroxime, cefpodoxime) were more frequently prescribed. However, from 2017 onward, these cephalosporins were no longer used as oral therapy, except in the final trimesters of the post-implementation period (Figure 4, Table S4).

### 3.4. DOT, LOT, and DOT/LOT

**Figure 5:**
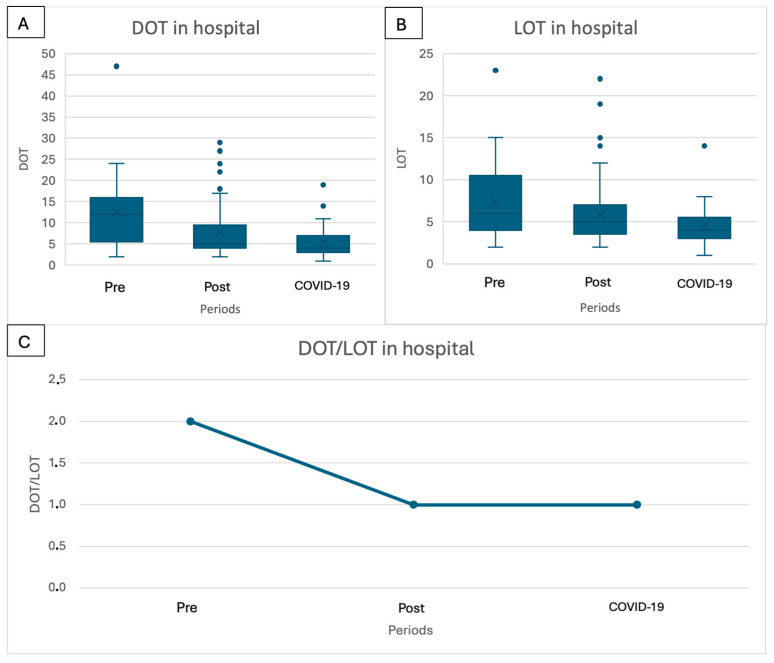
Days of therapy and length of therapy, considering antibiotics prescribed in hospital: (**A**) days of therapy; (**B**) length of therapy; (**C**) ratio between length of therapy and days of therapy.

Considering the antibiotic prescriptions in the hospital (both parenteral and oral), the median days of therapy and the median length of therapy declined when comparing the pre-implementation period to the post-implementation and the COVID-19 period, from 12 days to 5 days and 4 days (*p* < 0.001) and from 6 to 5 to 4 days (*p* = 0.003), respectively (Table 3, Figure 5A,B). The DOT/LOT ratio declined from 2 in the pre-implementation period to 1 in the post-implementation period (Figure 5C).

**Figure 6:**
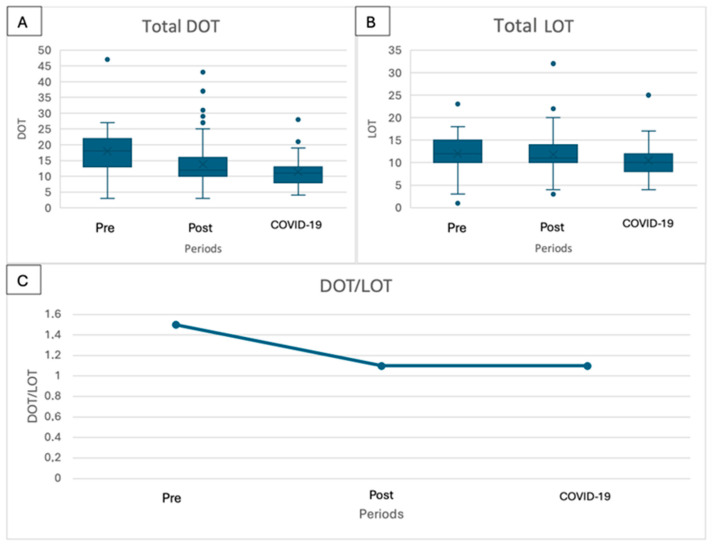
Days of therapy and length of therapy considering the total course of antibiotics for the treatment of skin and soft-tissue infection: (**A**) days of therapy; (**B**) length of therapy; (**C**) ratio between length of therapy and days of therapy.

A similar reduction was observed considering the total antibiotics prescribed (both in the hospital and at home at discharge). The days of therapy and length of therapy declined from 18 to 12 to 11 days (*p* < 0.001) and from 12 to 10 to 11 days (*p* = 0.073), respectively (Table 3, Figure 6A,B), and the DOT/LOT ratio declined from 1.5 in the pre-implementation period to 1.1 in the post-implementation and COVID-19 periods (Figure 6C).

### 3.5. Microbiological Results

**Figure 7:**
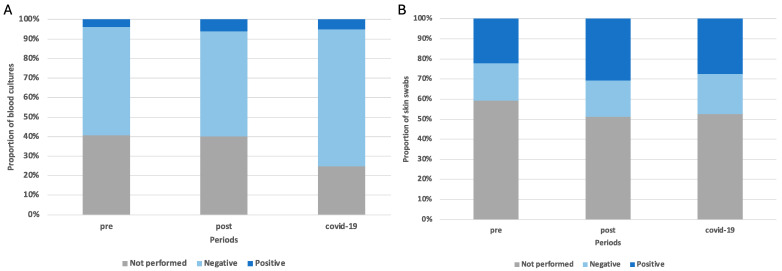
Microbiological tests performed in the three periods for children admitted with SSTIs: (**A**) blood cultures; (**B**) skin swabs.

A blood culture was performed for 116 patients (116/184, 63.0%), with rates of 59.3% (16/27) in the pre-implementation period, 59.8% (70/117) in the post-implementation period, and 75.0% (30/40) during the COVID-19 period (Figure 7A, Table 4). Only ten blood cultures were positive (10/184, 8.6%): two for *S. aureus*, one for *S. pyogenes*, two for *E. coli*, and the remainder for coagulase-negative staphylococci or other bacteria (Table 4).

Fewer than half of the children admitted had a skin swab or purulent material collection performed (87/184, 47.3%), with a positive rate of 57.5% (50/87). The proportion of skin swabs conducted was similar across the three study periods (Figure 5B, Table 3). The most commonly isolated bacterium was *S. aureus* (76.0%, 38/87) while other bacteria were less frequently found. An increase in *S. pyogenes* isolation was noted during the COVID-19 period compared to the other periods. Most *S. aureus* isolates from skin swabs were multisensitive (71.1%, 27/38); 15.8% (6/38) were resistant to methicillin (MRSA) but not to other different classes of antibiotics while 13.2% (5/38) were resistant to methicillin and other antibiotics, especially clindamycin. No significant changes in the types of *S. aureus* isolated were observed across the three periods (Table S5).

### 3.6. Safety and Other Clinical Outcomes

No adverse drug reaction related to antibiotic therapy was reported throughout the eight years.

The median length of stay decreased over time, from six days in the pre-implementation period to 5 days in the post-implementation period to 4 days in the COVID-19 period.

## 4. Discussion

Our study results demonstrate the efficacy and sustainability of a multifaceted antibiotic stewardship program in a pediatric acute care unit. CPs, educational talks, and the involvement of the pediatric infectious disease specialist during ward rounds were swiftly incorporated into the daily clinical routines of residents and senior physicians. Altogether, these interventions led to a reduction in the prescription of broad-spectrum antibiotics, particularly third-generation cephalosporins and glycopeptides, in children admitted with SSTIs. The use of Access antibiotics, both oral and parenteral, increased throughout the post-implementation period, surpassing 60% of total prescriptions, meeting the WHO-recommended thresholds for both inpatient and outpatient settings [12]. This finding aligns with other studies showing the efficacy of antibiotic stewardship interventions in pediatric settings [10,13,14].

The reduction in the use of third-generation cephalosporins and glycopeptides represents a significant achievement of the ASP as these antibiotics were commonly used before the program’s implementation. Although the retrospective nature of the study prevented us from assessing the severity of each patient’s condition upon admission, it is important to note that the ASP did not alter severity classifications or admission criteria; its focus was solely on improving antibiotic prescriptions. The updated recommendations and educational sessions effectively altered prescribers’ behavior, increasing their confidence in using narrower-spectrum antibiotics. Indeed, educational interventions have proven essential for achieving and sustaining positive results. Various studies from both Italy and the USA have shown that discontinuing educational initiatives or direct comparisons with the ASP team (audit and feedback) leads to a loss of previous gains [15,16,17].

The use of Reserve antibiotics was limited to one case involving a severe infection with a treatment failure of first-line therapy, for which linezolid was prescribed after consultation with pediatric infectious-disease specialists.

The analysis of DOT and LOT data demonstrated a statistically significant reduction in both metrics concerning the antibiotics prescribed during hospital admission, aligning with the observed decrease in the length of stay. Furthermore, the ratio between DOT and LOT decreased from two to one, underscoring the reduced use of combination therapy with ceftriaxone and glycopeptides. In the pre-implementation period, a majority of patients were treated with a combination of at least two antibiotics, resulting in a ratio of two. Conversely, during the post-implementation and COVID-19 periods, children were more frequently treated with only one antibiotic. The same analysis considering the total course of antibiotics for treating SSTIs, including both hospital and home treatment, revealed similar trends, although the difference in the LOT was not statistically significant. Notably, the overall duration of therapy for SSTIs declined slightly, indicating an opportunity for further improvement in the coming years.

Despite the increased use of Access antibiotics, there was no corresponding increase in the length of hospital stay. Indeed, the length of stay decreased over the study periods, likely due to a faster transition from parenteral to oral therapy and earlier discharge from the ward.

While antibiotic stewardship programs were largely disrupted worldwide during the COVID-19 pandemic, leading to a decline in appropriate antibiotic prescribing [18,19], our study found that the positive outcomes achieved during the post-implementation period were further improved during the COVID-19 period. This improvement occurred despite the reduction in the number of in-person antibiotic stewardship activities. However, it is important to note that during the COVID-19 pandemic, particularly in the first year, the number of admissions due to infectious diseases significantly decreased. School closures, physical distancing, hand hygiene, and other non-pharmacological interventions implemented during the pandemic not only curtailed the spread of SARS-CoV-2 but also reduced the transmission of other infectious diseases, particularly among children. As a result, the number of pediatric patients eligible for inclusion in our study was reduced.

Our internal guidelines recommended first-generation cephalosporins as the first-line treatment, both oral and parenteral; however, these antibiotics were less commonly prescribed in our study. This may be attributed to the limited availability of oral first-generation cephalosporins in Italy, leading physicians to prescribe amoxicillin-clavulanate upon discharge [20]. Indeed, children have faced an even greater shortage of oral therapies compared to adults, particularly during the pandemic and in the years that followed [21,22]. A major challenge for the pediatric population is the need for age-appropriate oral formulations [23]. Pre-dosed oral tablets are not suitable for younger children unless they have adequate body weight and the ability to swallow tablets. In many cases, tablets cannot be split to provide the correct dosage based on a child’s weight. Oral suspensions represent the preferred formulation for children, but their production was severely impacted by the pandemic.

Microbiological testing was not performed with all admitted children. Blood cultures were conducted for 60% of the children and yielded positive results in fewer than 10% of cases, consistent with other studies indicating a low probability of positive blood cultures in uncomplicated SSTIs [24,25]. Skin swabs were performed for about 50% of the children admitted, with a higher positive rate compared to blood cultures. Consistent with the literature, *S. aureus* was the most common pathogen isolated, with nearly 30% of strains being multidrug-resistant (MDR), including MRSA and strains resistant to other antibiotics such as clindamycin [1]. Although the rate of MRSA isolation in our study was similar to that reported in other studies [26], this percentage may have been overestimated compared to the real rate of MRSA in the community setting as children admitted to the ward with SSTIs could have had more severe diseases with higher rates of resistance compared to all children with SSTIs. The MRSA rate might have been lower if all children evaluated in the emergency department, including those not admitted, were considered.

While the indiscriminate use of antibiotics can lead to increased antibiotic resistance, improved prescribing practices have the potential to reduce the prevalence of multidrug-resistant bacteria [27]. Our study did not show differences in the types of bacteria responsible for infections over the eight-year period, possibly because the infections were acquired in the community. This suggests that efforts to improve antibiotic prescribing should also extend to ambulatory settings. 

A key strength of our study was its ability to assess not only the impact of the ASP but also its sustainability over an 8-year period, including the COVID-19 period. The ASP included recap lessons every two years to address the high turnover of physicians, particularly residents, in the emergency department and PACU. Although we cannot evaluate the efficacy of each specific intervention, consistent with our findings, we underscore the importance of educational sessions and recap lessons in achieving and maintaining effective antibiotic stewardship [17,28,29]. No other interventions, aside from those reported in the manuscript, were implemented in our department during the study period. These interventions were low-cost, allowing for the attainment of desired health outcomes while reducing unnecessary expenses and optimizing the use of resources. The intervention implemented in our setting may be easily replicable and applicable in similar settings within high-income countries.

This study presents some limitations. First of all, due to the retrospective nature of the study, it may be possible that we missed or misunderstood some of the information reported in the electronic medical records and it was not possible to have a 30-day follow-up after discharge. Secondly, despite the extended study period, data were collected from only 184 children. This relatively small sample size was used largely due to the nature of SSTIs, many of which are managed at home with topical treatments or oral antibiotics prescribed by primary care physicians without requiring hospital admission. Consequently, only children with moderate to severe infections were hospitalized, substantially limiting the number of participants in the study. Given that the majority of SSTIs are treated in the outpatient setting, it is essential to implement antimicrobial stewardship interventions in this context as well. Such initiatives can improve antibiotic prescribing practices and help mitigate the growing burden of AMR.

Although our study yielded promising results, further multicenter prospective studies implementing similar interventions in comparable settings are necessary to validate the impact of using ASPs on antibiotic prescriptions.

## 5. Conclusions

The implementation of a comprehensive, multifaceted antibiotic stewardship program proved to be effective in optimizing antibiotic use and reducing reliance on broad-spectrum antibiotics, with sustained benefits observed even during the COVID-19 pandemic. The increase in Access antibiotic prescriptions and the reduction in the use of third-generation cephalosporins and glycopeptides underscore the program’s success. Despite some limitations, including the relatively small sample size and the challenges associated with managing community-acquired infections, our study results highlight the importance of continuous education and adaptive strategies in maintaining effective antibiotic stewardship. Future efforts should focus on extending these practices to ambulatory settings to further improve antibiotic prescribing and combat antibiotic resistance.

## Figures and Tables

**Table 1:** Characteristics of the study population. Abbreviations: SSTI, skin and soft-tissue infection; LOS, length of hospital stay.

	Pre-Implementation Period	Post-Implementation Period	COVID-19 Period	Total	*p*
Patients with SSTIs (n)	27	117	40	184	-
Median age in months (25–75°)	41.0	58.4	54.6	54.1	0.99
	(28.1–107.0)	(18.2–111.0)	(16.0–108.0)	(18.6–109.4)	
Sex					
Male	17 (63.0%)	70 (59.8%)	23 (57.5%)	110 (59.8%)	0.90
Comorbidities					
0	22 (81.5%)	83 (71.0%)	32 (80.0%)	137 (74.5%)	0.34
≥1	5 (18.5%)	34 (29.0%)	8 (20.0%)	47 (25.5%)	
Median LOS in days (25–75°)	6 (4–9)	5 (4–7)	4 (3–5)	5 (4–7)	0.02
Children with ongoing oral antibiotic therapy at admission	7 (26.0%)	41 (35.0%)	13 (32.5%)	61 (33.1%)	0.65
Children transferred to other wards/hospital before the end of the treatment	2 (7.4%)	8 (6.8%)	3 (7.5%)	13 (7.1%)	0.99
Switch to oral from IV	19 (70.0%)	89 (76.0%)	29 (72.5%)	117 (63.6%)	0.78
During hospitalisation	3 (15.0%)	27 (30.0%)	10 (34.5%)	40 (21.7%)	0.34
At discharge	16 (85.0%)	62 (70.0%)	19 (65.5%)	97 (52.7%)	0.34

**Table 2:** Types of skin and soft-tissue infections.

Diagnosis	Pre-Implementation Period	Post-Implementation Period	COVID-19 Period	Total
Cellulitis	17 (63.0%)	63 (53.8%)	20 (50.0%)	100 (54.3%)
Skin abscess	4 (14.8%)	16 (13.7%)	2 (5.0%)	22 (11.9%)
Impetigo	3 (11.1%)	10 (8.5%)	5 (12.5%)	18 (9.8%)
Traumatic wound	1 (3.7%)	14 (12.0%)	4 (10.0%)	19 (10.3%)
Infection of surgical wound	0 (0%)	4 (3.4%)	2 (5.0%)	6 (3.3%)
Burns	0 (0%)	2 (1.7%)	4 (10.0%)	6 (3.3%)
Animal bites	0 (0%)	0 (0%)	2 (5.0%)	2 (1.1%)
Pyomyositis	1 (3.7%)	1 (0.8%)	0 (0%)	2 (1.1%)
Erysipelas	0 (0%)	2 (1.7%)	0 (0%)	2 (1.1%)
Fasciitis	0 (0%)	1 (0.8%)	0 (0%)	1 (0.5%)
Others	1 (3.7%)	4 (3.4%)	1 (2.5)	6 (3.3%)

**Table 3:** DOT, LOT, and DOT/LOT data for antibiotic treatments prescribed in hospital setting and for total antibiotics course for the treatment of SSTIs.

	Pre-Implementation Period	Post-Implementation Period	COVID-19 Period	*p* Value
Hospital antibiotic				
DOT (days and IQR)	12 (7–16)	5 (4–9)	4 (3–6.25)	<0.001
LOT (days and IQR)	6 (4–10.5)	5 (3–7)	4 (3–5)	0.03
DOT/LOT	2	1	1	
Total antibiotic course				
DOT (days and IQR)	18 (14–22)	12 (10–16)	11 (8–12)	<0.001
LOT (days and IQR)	12 (10–15)	11 (10–14)	10 (8–12)	0.073
DOT/LOT	1.5	1.1	11.1	

**Table 4:** Blood cultures and skin swabs of patients admitted with SSTIs.

	Pre-Implementation Period *n* = 27	Post-Implementation Period *n* = 117	COVID-19 Period *n* = 40	Total *n* = 184	*p* Value
Blood cultures performed	16 (59.3%)	70 (59.8%)	30 (75.0%)	116 (63.0%)	0.21
Positive	1 (6.25%)	7 (10%)	2 (6.7%)	10 (8.6%)	0.81
*S. aureus*	1 (100%)	1 (14.3%)	0	2 (20.0%)	
*S. pyogenes*	0	0	1 (50.0%)	1 (10.0%)	
*E. coli*	0	1 (14.3%)	1 (50.0%)	2 (20.0%)	
*Coagulase Negative Staphylococci*	0	3 (42.9%)	0	3 (30.0%)	
*Other bacteria*	0	2 (28.6%)	0	2 (20.0%)	
Skin swabs performed	11 (40.7%)	57 (48.7%)	19 (47.5%)	87 (47.3%)	0.76
Positive	5 (45.5%)	34 (59.7%)	11 (57.8%)	50 (57.5%)	0.68
*S. aureus*	4 (80.0%)	27 (79.4%)	7 (63.6%)	38 (76.0%)	
*S. pyogenes*	0	1 (2.9%)	4 (36.4%)	5 (10.0%)	
*E. coli*	1 (20.0%)	3 (8.8%)	0	4 (8.0%)	
*Other bacteria*	0	3 (8.8%)	0	3 (6.0%)	

## Data Availability

The data used in this study cannot be made publicly available due to Italian data protection laws. The anonymized datasets generated during the current study can be provided on request, from the corresponding author, after written approval by the local ethic committee.

## Supplementary Materials

The following supporting information can be downloaded at: https://www.mdpi.com/article/10.3390/children11111325/s1, Table S1. Antibiotic prescription in hospital stratified by AWaRe class and period; Table S2. Antibiotic prescription in hospital stratified by antibiotics and period; Table S3. Oral antibiotic prescription after parenteral therapy stratified by AWaRe class and period; Table S4. Oral antibiotic prescription after parenteral antibiotic therapy stratified by antibiotics and period; Table S5. Spectrum of resistance of *S. aurues* on skin swab.
