# Supplementary material for: Impact of Antibiotic Stewardship on Treatment of Hospitalized Children with Skin and Soft-Tissue Infections
Source: Children (Basel). 2024 Oct 30;11(11):1325. doi: 10.3390/children11111325 (PMC11593291; doi:10.3390/children11111325)
Supplement: Supplementary file 1 [file children-11-01325-s001.zip › children-3244293-supplementary.pdf]

Table S1. Antibiotic prescription in hospital stratified by AWaRe class and period.

	PRE-IMPLEMENTATION PERIOD	POST-IMPLEMENTATION PERIOD	COVID-19 PERIOD	TOTAL	p value
**Total prescriptions**	57	199	61	317	
**Access**	17 (30.0%)	129 (64.8%)	49 (80.0%)	195	<0.00001
**Watch**	40 (70.0%)	69 (34.7%)	12 (20.0%)	121	<0.00001
**Reserve**	0	1 (0.5%)		1	-

Table S2. Antibiotic prescription in hospital stratified by antibiotics and period.

	PRE-IMPLEMENTATION PERIOD	POST-IMPLEMENTATION PERIOD	COVID-19 PERIOD	TOTAL	p value
**Total prescriptions**	57	199	61	319	
**Aminopenicillin** (amoxicillin-clavulanic acid or ampicillin-sulbactam)	2 (3.5%)	70 (32.5%)	37 (60.7%)	109 (34.4%)	<0.00001
**Penicillin** (oxacillin)	4 (7.0%)	11 (5.5%)	1 (1.6%)	16 (5.0%)	0.36
**I gen cephalosporins** (cefazolin or cefalexin)	2 (3.5%)	8 (4.0%)	4 (6.6%)	14 (4.4%)	0.67
**III generation cephalosporins** (ceftriaxone, ceftazidime)	23 (40.4%)	41 (20.6%)	6 (9.8%)	70 (22.1%)	<0.001
**Glycopeptides** (vancomycin or teicoplanin)	12 (21.1%)	18 (9.0%)	1 (1.6%)	31 (9.8%)	0.002
**Lincosamides** (clindamycin)	1 (1.8%)	28 (14.1%)	5 (8.2%)	34 (10.7%)	0.023

**Other antibiotics**	13 (22.8%)	23 (11.6%)	7 (11.5%)	43 (13.6%)	0.08

Table S3. Oral antibiotic prescription after parenteral therapy stratified by AWaRe class and period

	PRE-IMPLEMENTATION PERIOD	POST-IMPLEMENTATION PERIOD	COVID-19 PERIOD	TOTAL	p value
**Total prescriptions**	16	82	30	128	
**Access**	10 (62.5%)	76 (92.7%)	30(100,0%)	116 (90.6%)	0.0004
**Watch**	6 (37.5%)	6 (7.3%)	0	12 (9.4%)	

Table S4. Oral antibiotic prescription after parenteral antibiotic therapy stratified by antibiotics and period.

	PRE-IMPLEMENTATION PERIOD	POST-IMPLEMENTATION PERIOD	COVID-19 PERIOD	TOTAL	p value
**Total prescriptions**	16	82	30	128	
**Aminopenicillin** (amoxicillin-clavulanic acid)	9 (56.3%)	59 (72.0%)	27 (90.0%)	95 (74.2%)	0.033
**I gen cephalosporins** (cefalexin)	0	5 (6.1%)	0	5 (3.9%)	
**II and III generation cephalosporins** (cefuroxime, cefpodoxime)	4 (25.0%)	4 (4.9%)	0	8 (6.3%)	
**Other antibiotics**	3 (18.8%)	14 (23.7%)	3 (10%)	20 (15.6%)	

Table S5. Spectrum of resistance of *S. aurues* on skin swab.

**Skin swabs**	**PRE-IMPLEMENTATION PERIOD N = 27**	**POST-IMPLEMENTATION PERIOD N = 117**	**COVID-19 PERIOD N = 40**	**TOTAL N = 184**	**p value**
**Positive**	5 (45.5%)	34 (59.7%)	11 (57.8%)	50 (57.5%)	
S. aureus	4 (80.0%)	27 (79.4%)	7 (63.6%)	38 (76.0%)	
MSSA	2 (50.0%)	20 (74.1%)	5 (71.4%)	27 (71.1%)	0.61
MRSA	2 (50.5%)	3 (11.1%)	1 (14.3%)	6 (15.8%)	0.14
MDR	0	4 (14.8%)	1 (14.3%)	5 (13.2%)	
